# Association of vitreous vitamin C depletion with diabetic macular ischemia in proliferative diabetic retinopathy

**DOI:** 10.1371/journal.pone.0218433

**Published:** 2019-06-19

**Authors:** Sung Wook Park, Woonhyung Ghim, Sanghyeon Oh, Yejin Kim, Un Chul Park, Jaeseung Kang, Hyeong Gon Yu

**Affiliations:** 1 Department of Ophthalmology, Seoul National University College of Medicine, Seoul, Republic of Korea; 2 Department of Ophthalmology, Seoul National University Hospital, Seoul, Republic of Korea; 3 Retinal Degeneration Laboratory, Biomedical Research Institute, Seoul National University Hospital, Seoul, Republic of Korea; 4 Interdisciplinary Program in Stem Cell Biology, Seoul National University College of Medicine, Seoul, Republic of Korea; 5 Department of Anatomy, Seoul National University College of Medicine, Seoul, Republic of Korea; Boston University School of Medicine, UNITED STATES

## Abstract

**Purpose:**

Vitreous vitamin C, as an anti-oxidant, is responsible for regulating oxygen tension and oxidative stress in the eye. Oxidative stress and retinal ischemia are implicated in the development of proliferative diabetic retinopathy (PDR). In this study, we aimed to determine whether vitreous level of vitamin C is compromised in patients with PDR and to investigate the association of diabetic macular ischemia and vitamin C.

**Methods:**

This prospective study enrolled forty patients who underwent pars plana vitrectomy for the treatment of PDR (PDR group, n = 20) and idiopathic epiretinal membrane (control group, n = 20). Serum, aqueous humor, and the vitreous were collected for the analysis of vitamin C level by HPLC. Diabetic macular ischemia (DMI) in PDR group was evaluated with fluorescein angiography (FA).

**Results:**

PDR patients (60.4 ± 2.1 y) were younger than non-diabetic control patients (67.4 ± 1.2 y). Serum, aqueous, and vitreous levels of vitamin C in PDR were 38.7%, 22.5%, and 11.1% of non-diabetic control group, respectively. All PDR patients had DMI (grade 1: 25%, grade 2: 30%, grade 3: 30%, grade 4: 15%). DMI grade was inversely correlated with the level of vitreous vitamin C (r = -0.546, P = 0.019), not with HbA1C, serum, or aqueous vitamin C level. In addition, the level of vitreous vitamin C (4.5 ± 2.6 μg/ml) in high DMI group (Gr 3 &4) was lower than that (31.0 ± 9.1 μg/ml) in low DMI group (Gr 1&2) (*P* = 0.015).

**Conclusions:**

Vitreous level of vitamin C in PDR patients showed a tenfold decrease, which was associated with the degree of macular ischemia. This suggests that vitreous vitamin C depletion may cause macula ischemia in PDR patients.

## Introduction

Diabetic retinopathy (DR) is the most common microvascular complication of diabetes mellitus and is a leading cause of blindness in the working population worldwide [1]. Both diabetic macular edema and proliferative diabetic retinopathy (PDR) cause severe visual impairment [2]. Diabetic macular ischemia (DMI) is an important feature of DR, characterized by occlusion and loss of the macular capillary network (capillary dropout) [3]. Hyperglycemia and retinal ischemia are the main causes of macular edema and neovascularization in PDR. According to the Early Treatment of Diabetic Retinopathy Study (ETDRS) report 11, DMI assessed by fluorescein angiography (FA), in a clinical setting, is correlated with poor visual prognosis [3]. In addition, DMI is associated with PDR [4].

In DR, hypoxia-induced oxidative stress plays an important role in ischemic retinopathy [5]. Metabolic mechanisms are associated with excess production of reactive oxygen species (ROS) and depletion of antioxidants in DR [6]. Vitamin C (ascorbic acid), a water-soluble vitamin, functions diversely in the body as an anti-oxidant [7]. In patients with diabetes, the serum vitamin C level is low [8]. Additionally, reduced serum vitamin C levels have been reported in type 2 diabetes patients with DR as compared to those in type 2 diabetic patients without DR [9]. In another study, the vitamin C intake did not differ between type 2 diabetic patients and control subjects, yet the serum vitamin C levels were found to be low in type 2 diabetic patients as compared to those in the control subjects [10].

The concentration of vitamin C in the human vitreous humor is remarkably high as compared to that in serum [11]. However, vitreous vitamin C is low in patients with PDR [12]. Yet, the association of diabetic macular ischemia with vitreous vitamin C has not been investigated to date. We conjectured that low vitamin C in the vitreous humor may be the result of impaired accumulation or excessive consumption of vitamin C due to oxidative stress in ischemic retinopathy such as PDR. This low level of vitamin C in the vitreous humor can, in turn, cause ischemia-induced oxidative stress in PDR. Thus, we hypothesized that retinal ischemia is associated with vitreous vitamin C depletion in PDR. In this study, we have determined whether vitreous vitamin C is decreased in patients with PDR. In addition, we investigated the association between DMI and vitamin C in PDR. We also analyzed the correlation of vitamin C level among the serum, aqueous humor, and vitreous humor in patients with PDR.

## Methods

This prospective study was approved by the institutional review board of the Seoul National University Hospital (IRB No. 1604-065-754). The study followed Basics of Good Clinical Practice and the tenets of the Helsinki declaration and its later amendments or comparable ethical standards. All participants provided informed consent after receiving an explanation of the nature and possible consequences of the study.

### Patients

Patients over 40 years old were enrolled in this study. Any subject who smoked, took multi-vitamins or nonsteroidal anti-inflammatory agents, or hormone replacement therapy was ineligible for the study. In addition, any patient with a history of macrovascular disease was excluded. Only patients who were not taken vitamin C supplement for 6 months prior to surgical sampling was included. PDR was diagnosed with fluorescein angiography (FA) for new vessels or any evidence of vitreous hemorrhage, traction retinal detachment. Only non-diabetic patients with idiopathic epiretinal membrane were enrolled as a control group.

### Sample collection

Blood samples for serum analysis (5 ml) were simultaneously collected from the cubital vein, in vials containing EDTA, and were centrifuged at 1200 g at 4°C. Serum samples were stored at -80°C. Aqueous humor samples (0.05–0.1 ml) were rapidly collected at the beginning of the surgical procedure through a limbal paracentesis of 1 mm at 12 o’clock direction with a 26-gauge needle on a tuberculin syringe. Special care was taken to avoid collapsing the anterior chamber. Samples were transferred to marked brown-colored microcentrifuge tube and immediately stored at liquid nitrogen. Vitreous humor samples (0.05–0.1 ml) were obtained by the standard vitreoretinal aspiration procedure, pars plana vitrectomy before infusion started. Samples were placed in marked brown-colored microcentrifuge tube and immediately stored at liquid nitrogen. All procedures were conducted by single surgeon (H.G.Y.).

### Vitamin C analysis

Vitamin C level in the samples was determined by measuring ascorbic acid using high-performance liquid chromatography (HPLC) technique. The equipment used for the procedure are composed of UV lamp (D2 LAMP, Schambeck, Bad Honnef, Germany), Column (ProntoSIL 120-5-C18SH, 5 μm, 150X4.6 mm, Bischoff, Leonberg, Germany), UV detector (NS2100D, FUTECS, Daejeon, South Korea), HPLC column oven (AT4000, FUTECS, Daejeon, South Korea), solvent delivery system (NS-2004GP, FUTECS, Daejeon, South Korea) and HPLC vacuum degasser (ERC-3415a, ERC Inc, AL, USA). Human aqueous humor, vitreous and serum samples in -70°C freezer were thawed in 4°C fridge and 50 μl of each sample was mixed with 50 μl 10% meta-phosphoric acid (meta-phosphoric acid, Sigma Aldrich, Missouri, USA) in HPLC water and reacted on ice for 30 minutes. After the reaction, tubes were centrifuged for 5 minutes with 14,000 rpm. Supernatant in each tube was isolated and used for the chromatography. 50 μl of each sample was injected into the chromatograph when analyzed. Mobile phase for the HPLC was made as following. First, 14 g monochloroacetic acid, 4.5 g solid sodium hydroxide, 1 ml OSA solution (30mg/ml octane sulfonic acid in HPLC water) and 0.75 g EDTA were added in 950 ml HPLC water. All reagents were obtained from Sigma Aldrich. Then pH of the solution was set to 3.0 by adding HCl. After adding 50 ml HPLC methanol (Methanol for HPLC, J.T. Baker Chemicals, NJ, USA), the solution was filtered using 0.22 μm filter. Then the bottle with the solution was sonicated for 5 minutes to remove any residing bubbles. Standard curve for ascorbic acid was obtained by measuring 8 different concentrations of ascorbic acid (L-Ascorbic acid, Sigma Aldrich, Missouri, USA) dissolved in HPLC water (HPLC grade water, J.T. Baker Chemicals, NJ, the USA). First, 125 μg/ml of standard solution was made. Then it was serially diluted in half each time for 6 times. With these 7 different concentrations of solution and HPLC water alone, total of 8 different standard solutions were injected into the chromatograph with 50 μl portion, respectively.

### Assessment of diabetic macular ischemia with fluorescein angiograms

Diabetic macular ischemia (DMI) was graded by 2 masked ophthalmologists in agreement using protocols and standard photographs from ETDRS Report No. 11 [3, 13, 14]. According to these criteria, diabetic macular ischemia was classified as none (0), questionable (1), mild (2), moderate (3), or severe (4) (S1 Fig). All angiographic images was acquired with a digital retinal camera system (Topcon TRC 50IX; Topcon Medical Systems Inc, Paramus, New Jersey, USA). One early-to mid-phase image (at 20–40 seconds), centered on the macula, was chosen for analysis.

### Statistical analysis

Statistical methods were used to predetermine sample size. All group results were expressed as mean ± SEM, if not stated otherwise. Comparisons between groups were made using the two-tailed Student’s t-test for continuous variables and chi-square test for non-continuous variables. We compared vitamin C level after adjusting covariates of age and sex. Statistical significance as compared to control group was denoted with * (P < 0.05), ** (P < 0.01), *** (P < 0.001) in the tables. Statistical analysis was performed using IBM SPSS statistics 22 (IBM, Armonk, NY, USA).

## Results

Forty eyes of 40 patients underwent vitreous and aqueous humor sampling at the time of vitrectomy surgery. A total number of 20 eyes that were categorized as “proliferative diabetic retinopathy (PDR)” had ischemia-related retinal disease conditions (proliferation, vitreous hemorrhage, traction retinal detachment). The eyes categorized as “control” had idiopathic epiretinal membrane without evidence of another retinal disease such as a retinal tear, uveitis, or retinal vein occlusion. Demographic characteristics of patients are shown in Table 1. The mean age of the PDR group (60.4 ± 2.1 years) was lesser than that of the control group (67.4 ± 1.2 years). There was no significant difference in sex and in the presence of hypertension between the PDR group and non-diabetic control group.

**Table 1:** Demographic characteristics of patients in this study.

Characteristic	Non-diabetic control	Proliferative diabetic retinopathy	*P*
N	20	20	
Age, mean ± SD, y	67.4 ± 1.2	60.4 ± 2.1	**0.006**
Sex (M:F)	4: 16	9: 11	0.176
Hypertension (%)	12 (60.0)	7 (35.0)	0.205

Student’s t-tests or χ2 tests

**Fig 1:**
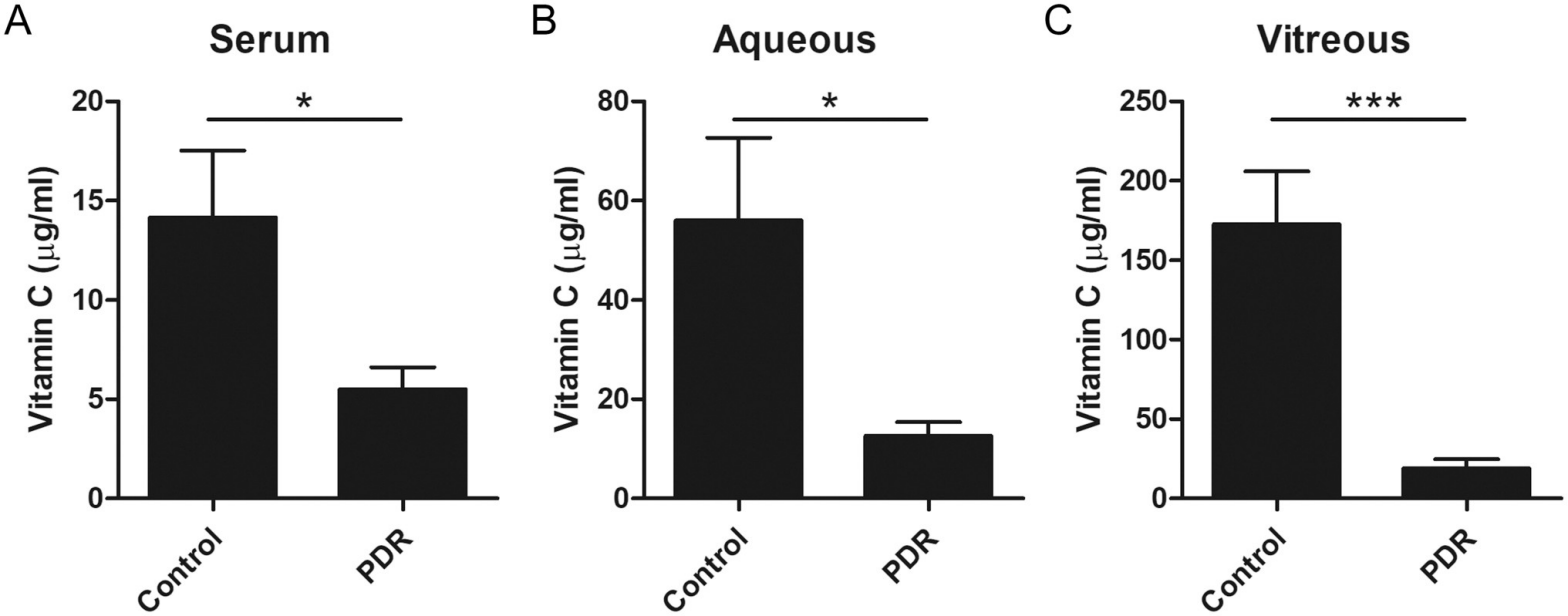
Comparison of vitamin C level in serum (A), aqueous humor (B), and vitreous humor (C) between patients with diabetic retinopathy and controls. Vitamin C (μg/ml), Student’s t-test, * P < 0.05, ***P < 0.001.

Vitamin C levels in the serum, aqueous humor, and vitreous humor were compared between PDR and control groups (Table 2 and Fig 1). Vitamin C in the vitreous humor was significantly lower in the PDR group (19.1 ± 5.8 μg/ml) as compared to that in the control group (172.7 ± 33.4 μg/ml, *P* < 0.001). In addition, vitamin C levels in the serum and aqueous humor were also lower in PDR group (serum: 5.5 ± 1.1, aqueous humor: 12.6 ± 2.8 μg/ml) as compared to the control group (serum: 14.2 ± 3.4, aqueous humor: 56.0 ± 16.7 μg/ml) (*P* = 0.023 and *P* = 0.019, respectively). After adjusting for age and sex, these low vitamin C levels in PDR were still statistically significant. Vitamin C level in PDR was 38.7% in serum, 22.5% in the aqueous humor, and 11.1% in the vitreous humor of non-diabetic control group.

**Table 2:** Comparison of vitamin C level in serum, aqueous humor, and vitreous humor between patients with diabetic retinopathy and controls.

	Non-diabetic control	Proliferative diabetic retinopathy	*P*^†^	*P*^‡^
N	20	20		
Ascorbate (μg/ml)				
Serum	14.2 ± 3.4	5.5 ± 1.1	**0.023**	**0.040**
Aqueous humor	56.0 ± 16.7	12.6 ± 2.8	**0.019**	**0.037**
Vitreous	172.7 ± 33.4	19.1 ± 5.8	**<0.001**	**<0.001**

^†^Student’s t-test,

^‡^Univariate ANOVA, adjusted by covariates: age, sex

**Fig 2:**
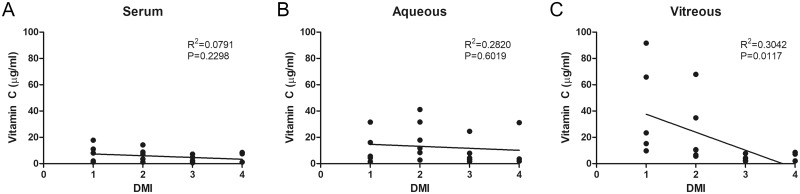
Correlation of diabetic macular ischemia (DMI) and vitamin C level in serum (A), aqueous humor (B), and vitreous humor (C) in patients with proliferative diabetic retinopathy.

In this study, all PDR patients had DMI (grade 1: 25%, grade 2: 30%, grade 3: 30%, grade 4: 15%). DMI was significantly correlated with vitreous vitamin C (r = -0.546, *P* = 0.019, Fig 2C). This correlation was statistically significant even after adjustment for age and sex (r = -0.552, *P* = 0.012). However, there was no correlation either between DMI and serum vitamin C (r = -0.281, *P* = 0.230, Fig 2A) or between DMI and aqueous vitamin C (r = -0.124, *P* = 0.602, Fig 2B). Vitreous vitamin C was low in patients of the high-DMI group (grades 3 and 4) as compared to that in patients of the low-DMI group (grades 1 and 2) (Table 3). In binary logistic regression analysis for the low- and high-DMI groups, the odds ratio of the vitreous vitamin C level was 0.531 (95% CI: 0.285–0.991, *P* = 0.047). These results suggest that oxidative stress in ischemic retinopathy, as well as the depletion of anti-oxidants, contributes to the pathogenesis of PDR.

**Table 3:** Comparison of vitamin C level in serum, aqueous humor, and vitreous humor according to diabetic macular ischemia severity in patients with proliferative diabetic retinopathy.

DMI severity	Grade 1 & 2	Grade 3 & 4	*P*^†^
	N = 11	N = 9	
Age	59.8 ± 7.7	61.1 ± 11.3	0.775
HbA1c (%)	8.1 ± 1.3	8.4 ± 1.3	0.639
Serum	6.9 ± 1.7	3.8 ± 1.1	0.166
Aqueous humor	15.7 ± 4.1	8.8 ± 3.7	0.241
Vitreous	31.0 ± 9.1	4.5 ± 2.6	**0.015**

Ascorbate (μg/ml), Student’s t-test

In the PDR group, 70% of the eyes had a history of panretinal photocoagulation, 70% showed neovascularizations on FA, 60% had traction membranes, and 50% had vitreous hemorrhage. However, in the subgroup analysis of the PDR group, there were no differences in vitamin C levels in the serum, aqueous humor, and vitreous humor according to the above mentioned ocular factors (S1 Table).

**Fig 3:**
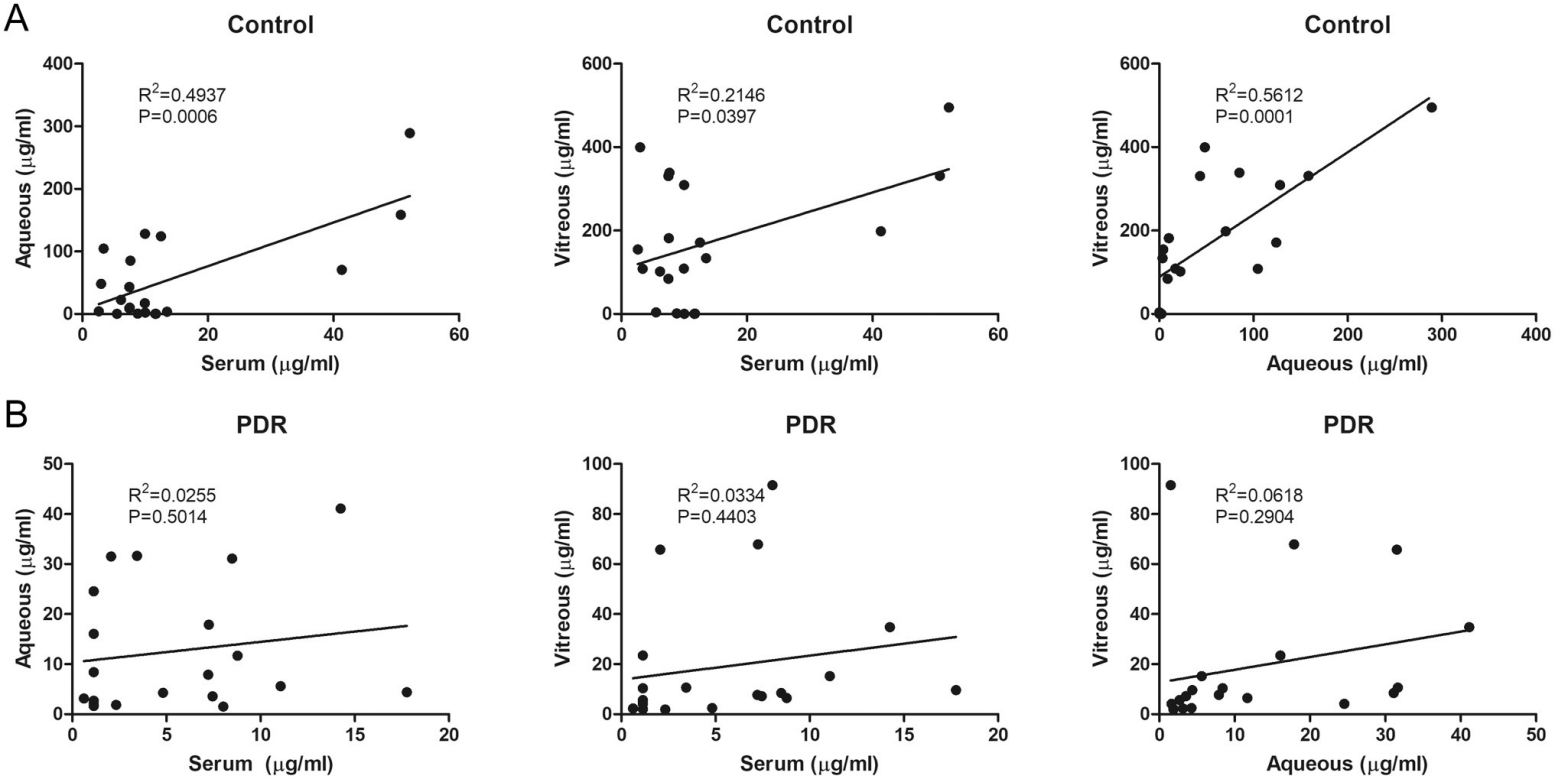
Correlation of vitamin C level in serum, aqueous humor, and vitreous humor between controls (A) and patients with proliferative diabetic retinopathy (B).

In the correlation analysis, the serum, aqueous humor, and vitreous humor levels of vitamin C were all significantly correlated in non-diabetic patients. However, there was no correlation in the serum, aqueous humor, and vitreous humor level of vitamin C in the PDR group (Table 4 and Fig 3).

**Table 4:** Correlation of vitamin C level in serum, aqueous humor, and vitreous humor between controls and patients with proliferative diabetic retinopathy.

	Non-diabetic control (N = 20)		Proliferative diabetic retinopathy (N = 20)	
	Pearson correlation coefficient	*P*^†^	Pearson correlation coefficient	*P*^†^
Ascorbate level				
Serum Aqueous	0.703	**0.001**	0.160	0.501
Serum Vitreous	0.463	**0.040**	0.183	0.440
Aqueous Vitreous	0.749	**<0.001**	0.249	0.290

^†^Pearson correlation analysis

## Discussion

In this study, we demonstrated that the levels of vitamin C in the serum, aqueous humor, and vitreous humor were lower in patients with PDR than those in non-diabetic patients. In addition, correlations of vitamin C levels in the serum, aqueous humor, and vitreous humor were not found to be significant in patients with PDR. Most importantly, we demonstrated that DMI was associated with vitreous vitamin C depletion in patients with PDR. DMI is a good biomarker for ischemia in DR as well as in the peripheral non-perfusion area. Since only PDR patients were included in this prospective study, prior history of laser photocoagulation impaired direct comparison of the peripheral ischemic area as an ischemic biomarker. Instead, we revealed, for the first time that DMI was associated with vitreous vitamin C depletion in PDR. In a previous study, researchers noted differential upregulations of various angiogenic growth factors such as vascular endothelial growth factors, angiopoietins, and various inflammatory cytokines (TNF-α, IL-1β, and IL-6) in the vitreous humor of PDR patients [15]. This implied that ocular factors, especially the retina and vitreous humor, are more important and relevant to the development of PDR than systemic factors.

Vitreous vitamin C is physiologically high in the non-diabetic eye whereas it is severely impaired in the diabetic eye. The mean concentration of ascorbate in the vitreous humor is approximately 2 mM, whereas blood levels are only 50–60 μM, which is a 33- to 40-fold difference [11]. The high concentration of vitamin C in the vitreous humor indicated that vitamin C acts as an anti-oxidant under high oxidative stress and has a protective effect on the retina, even in physiologically normal conditions. In this study, vitamin C level in PDR measured only 11.1% of vitreous vitamin C levels of the non-diabetic control group. This result suggested that the retina of patients with PDR are under significant oxidative stress, and that lower concentrations of vitreous vitamin C is associated with the pathogenesis of PDR. Several possibilities can explain the reduced level of vitamin C in the vitreous humor of PDR patients, which have been outlined below.

First, systemic vitamin C might be consumed more often by diabetic patients as compared to the control patients. Serum vitamin C level is known to be low in patients with diabetes [8]. Sinclair *et al*. reported that the concentration of ascorbic acid was decreased in the plasma of diabetic patients, particularly in those with diabetic complications [16]. In patients with type 2 diabetes, serum vitamin C is lower in patients with DR as compared to that in patients without DR [9]. Sundaram *et al*. showed that the levels of serum vitamin C gradually decreased in diabetes when the severity and number of secondary angiopathic complications increased [17]. Our findings are largely consistent with those of earlier studies.

Second, retinal ischemia might be related to vitreous vitamin C depletion in patients with PDR. In this condition, vitreous vitamin C gets consumed faster to compensate for the increased oxidative stress that is produced on the ocular tissues. Recently, Shui *et al*. found that the vitreous humor metabolizes molecular oxygen in an ascorbate-dependent manner, thereby regulating intraocular oxygen tension [18]. The vitreous humor is also important in the development of cataract and primary open angle glaucoma [19]. In this study, DMI, as an ischemic marker, was associated with vitreous vitamin C depletion in PDR patients. In addition, correlations between vitamin C levels in the serum, aqueous humor, and vitreous humor were not significant in patients with PDR. However, vitreous vitamin C was significantly decreased in patients with PDR. These results implied that ocular factors, especially retinal ischemia, are more prevalent than any systemic factors in decreasing vitreous vitamin C in PDR patients.

Another explanation states that there is an impaired accumulation of vitreous vitamin C in eyes with PDR. Similar to our study, Takano S *et al*. reported that vitamin C level of vitreous in PDR patients was lower than that of epiretinal membrane patients [12]. The high level of vitamin C in the vitreous humor is maintained by a sodium-dependent ascorbate transporter (SLC23A2) located in the pigmented layer of the ciliary epithelium [20]. Thus, in PDR patients, SLC23A2 expression might be suppressed, which may subsequently lead to impaired accumulation of vitreous vitamin C.

Vitamin C supplements can increase the plasma vitamin C level but cannot lower the blood glucose of diabetic patients [21]. Dietary intake of nutrients forms a crucial aspect of DR prevention and management. A recent systematic review that investigated the association between dietary intake and DR demonstrated that dietary fiber, oily fish, and a Med diet are protective against DR, while a higher caloric intake was associated with greater risk of DR [22]. Regarding vitamin C, Tanaka *et al*. [23] reported a protective relationship between vitamin C intake and incidence of DR (Q4 vs. Q1, HR, 95% CI: 0.61, 0.39–0.96), in contrast to a cross-sectional study by Mayer-Davis *et al*. [24] that reported a risk association between vitamin C intake and prevalence of DR (9th decile vs. 1st quintile, Odds Ratio [OR]: 2.21, *P* = 0.01). Till date, no significant associations have been observed between the risk of retinopathy and intake of major dietary antioxidants [25]. Experimentally, vitamin C was found to reduce retinal oxidative stress and prevent ultrastructural alterations in a porcine model [26]. The potential role of vitamin C in the treatment of DR remains open to debate, and it is suggested that future research focusing on patient‐oriented outcomes should address this important issue [27]. Thus, further prospective studies are warranted to elucidate whether vitamin C supplements can increase vitreous vitamin C level and protect the retina against ischemia in PDR patients.

In a previous study, the amount of vitamin C in the aqueous humor of patients with age-related cataract was found to decrease with age [28]. Although we did not observe an age-related decrease of vitamin C in the serum, aqueous humor, and vitreous humor, we adjusted both age and sex as a confounding factor in the analysis. Our findings regarding the serum level of vitamin C are comparable to previous studies [11, 29].

It is interesting to note that the levels of vitamin C are significantly correlated among the serum, aqueous humor, and vitreous humor in the control group. The compromised vitreous vitamin C level in PDR patients can be due to impaired transport of vitamin C or its excessive consumption in the vitreous humor. As a result, the vitreous vitamin C level in PDR patients was only 11.1% of the vitreous vitamin C levels of the non-diabetic control group as compared to 38.7% of serum vitamin C and 22.57% of aqueous vitamin C.

The depletion of vitamin C is considered a marker for oxidative stress in diabetes [30]. This differential suggests that the retina in patients with PDR may suffer from significant oxidative stress. Endotoxin-induced ocular inflammation caused a decrease in the concentration of ascorbic acid in the aqueous humor and an increase in the vitreous humor. The additional lack of correlation between levels of ascorbic acid in the physiologically normal aqueous and vitreous humors from the same uninflamed eye indicates that the aqueous humor is not the source of vitreous ascorbic acid [31]. In this study, there was no correlation among the serum, aqueous humor, and vitreous humor levels of vitamin C in the PDR group. This implied that although diabetes itself is a systemic disease, ocular factors are more important in the pathogenesis of PDR.

This study has a few limitations. First, we could not determine the causal relationship between vitreous vitamin C depletion and DMI in PDR patients. To investigate this further, we need to determine whether vitamin C supplements reduce DMI or whether vitamin C depletion aggravates DMI. We postulated that both vitreous vitamin C depletion and DMI participate in permanent loss of retinal neurons. The second was that there were very few participants in the subgroup analysis of PDR based on DMI severity.

In this study, we provided direct evidence of compromised vitamin C in the vitreous humor of patients with PDR. In the PDR group, DMI grade was inversely correlated with the level of vitreous vitamin C, not with HbA1C, serum, or aqueous vitamin C level. The results suggest that vitreous vitamin C depletion may cause macula ischemia in PDR patients.

## Supporting information

S1 Fig Representative images of diabetic macular ischemia in fluorescein angiography. Questionable (grade 1), mild (grade 2), moderate (grade 3), or severe (grade 4). White circle indicates inner ETDRS ring (1 disc diameter).
(TIF)

S1 Table Comparison of vitamin C level in serum, aqueous humor, and vitreous humor according to ocular factors in patients with proliferative diabetic retinopathy. (DOCX)

S2 Table Dataset of the study patients. (XLSX)
