# Supplementary figures and images for: Association of vitreous vitamin C depletion with diabetic macular ischemia in proliferative diabetic retinopathy
Source: PLoS One. 2019 Jun 19;14(6):e0218433. doi: 10.1371/journal.pone.0218433 (PMC6583975; doi:10.1371/journal.pone.0218433)

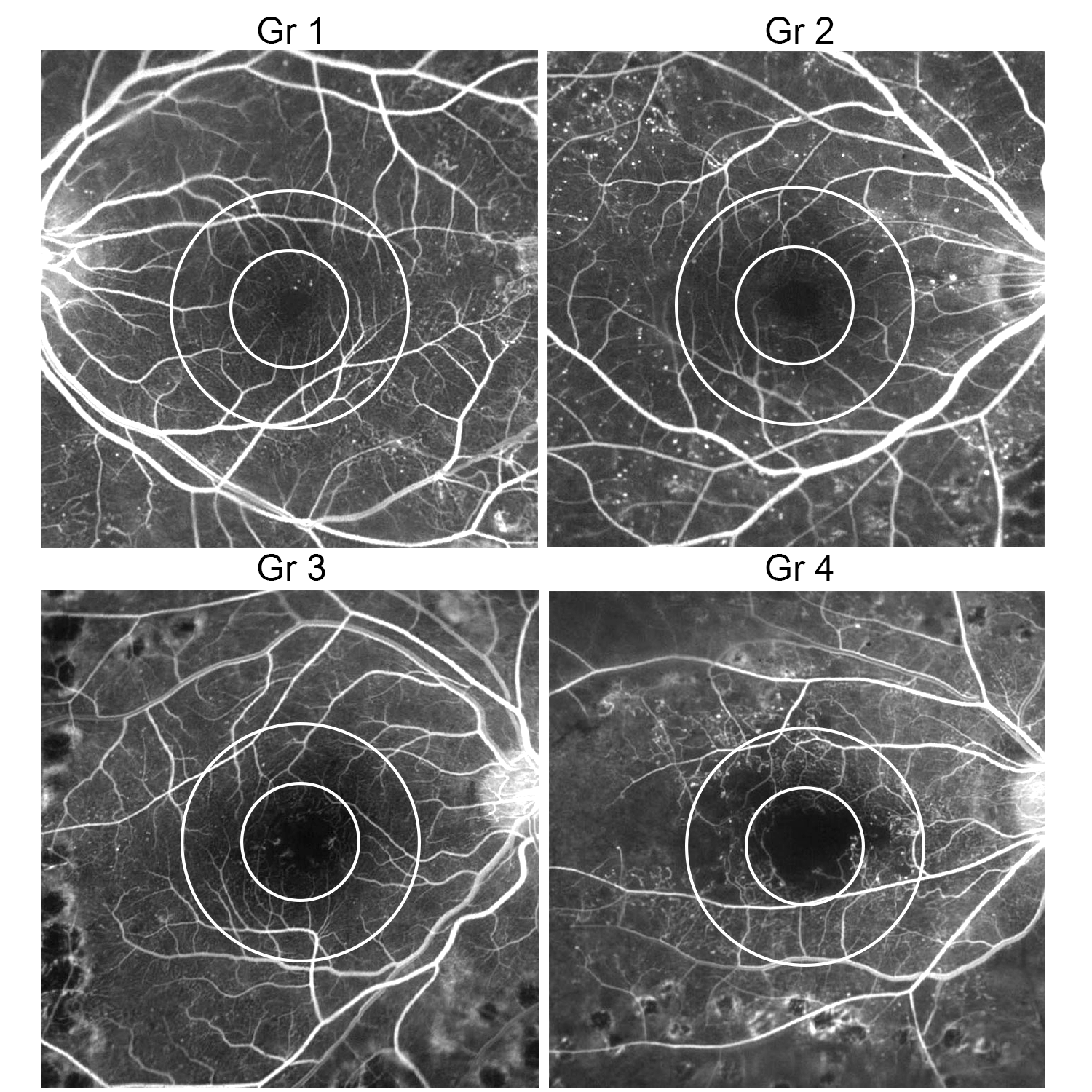

Supplement: S1 Fig — Questionable (grade 1), mild (grade 2), moderate (grade 3), or severe (grade 4). White circle indicates inner ETDRS ring (1 disc diameter). (TIF) [file pone.0218433.s001.tif]
