# Supplementary material for: Association of vitreous vitamin C depletion with diabetic macular ischemia in proliferative diabetic retinopathy
Source: PLoS One. 2019 Jun 19;14(6):e0218433. doi: 10.1371/journal.pone.0218433 (PMC6583975; doi:10.1371/journal.pone.0218433)
Supplement: S1 Table — (DOCX) [file pone.0218433.s002.docx]

| S1 Table. Comparison of vitamin C level in serum, aqueous humor, and vitreous humor according to ocular factors in patients with proliferative diabetic retinopathy | | | |
| --- | --- | --- | --- |
|  | - | + | *P* |
| **Prior PRP (N)** | 6 | 14 |  |
| Serum | 2.4 ± 1.1 | 6.9 ± 5.1 | 0.060 |
| Aqueous humor | 8.1 ± 4.0 | 14.5 ± 3.6 | 0.310 |
| Vitreous | 21.2 ± 14.5 | 18.2 ± 5.9 | 0.814 |
| **NVD or NVE (N)** | 6 | 14 |  |
| Serum | 3.4 ± 1.4 | 6.4 ± 1.4 | 0.219 |
| Aqueous humor | 7.0 ± 3.2 | 15.0 ± 3.7 | 0.118 |
| Vitreous | 31.9 ± 15.8 | 13.6 ± 4.6 | 0.310 |
| **Membrane (N)** | 8 | 12 |  |
| Serum | 5.0 ± 1.4 | 5.9 ± 1.6 | 0.693 |
| Aqueous humor | 14.8 ± 4.6 | 11.26 ± 3.7 | 0.540 |
| Vitreous | 22.3 ± 9.8 | 16.9 ± 7.4 | 0.662 |
| **Vitreous hemorrhage (N)** | 10 | 10 |  |
| Serum | 6.1 ± 1.9 | 5.0 ± 1.3 | 0.625 |
| Aqueous humor | 11.6 ± 4.5 | 13.6 ± 4.7 | 0.730 |
| Vitreous | 23.4 ± 10.0 | 14.8 ± 6.0 | 0.474 |
| Ascorbate (μg/ml), Student’s t-test | | | |
